# The voltage-gated K^+^ channel Kv1.3 modulates platelet motility and α_2_β_1_ integrin-dependent adhesion to collagen

**DOI:** 10.1080/09537104.2021.1942818

**Published:** 2021-08-04

**Authors:** Joy R Wright, Sarah Jones, Sasikumar Parvathy, Leonard K Kaczmarek, Ian Forsythe, Richard W Farndale, Jonathan M Gibbins, Martyn P Mahaut-Smith

**Affiliations:** 1 Department of Cardiovascular Sciences, University of Leicester, Leicester, UK; 2 Department of Molecular and Cell Biology, University of Leicester, Leicester, UK; 3 Department of Life Sciences, Manchester Metropolitan University, Manchester, UK; 4 Institute for Cardiovascular and Metabolic Research, University of Reading, Reading, UK; 5 Department of Cellular and Molecular Physiology, Yale University School of Medicine, USA; 6 Department of Neuroscience, Psychology and Behaviour, University of Leicester, Leicester, UK; 7 Department of Biochemistry, University of Cambridge, Cambridge, UK

## Abstract

Kv1.3 is a voltage-gated K^+^-selective channel with roles in immunity, insulin-sensitivity, neuronal excitability and olfaction. Despite being one of the largest ionic conductances of the platelet surface membrane, its contribution to platelet function is poorly understood. Here we show that Kv1.3-deficient platelets display enhanced ADP-evoked platelet aggregation and secretion, and an increased surface expression of platelet integrin α_IIb_. In contrast, platelet adhesion and thrombus formation *in vitro* under arterial shear conditions on surfaces coated with collagen were reduced for samples from Kv1.3^−/-^ compared to wild type mice. Use of collagen-mimetic peptides revealed a specific defect in the engagement with α_2_β_1_. Kv1.3^−/-^ platelets developed significantly fewer, and shorter, filopodia than wild type platelets during adhesion to collagen fibrils. Kv1.3^−/-^ mice displayed no significant difference in thrombus formation within cremaster muscle arterioles using a laser-induced injury model, thus other pro-thrombotic pathways compensate *in vivo* for the adhesion defect observed *in vitro*. This may include the increased platelet counts of Kv1.3^−/-^ mice, due in part to a prolonged lifespan. The ability of Kv1.3 to modulate integrin-dependent platelet adhesion has important implications for understanding its contribution to normal physiological platelet function in addition to its reported roles in auto-immune diseases and thromboinflammatory models of stroke.

## Introduction

Kv1.3 is a ubiquitously-expressed voltage-gated K^+^ channel with recognized roles in several physiological responses, including T cell activation, olfaction, and peripheral insulin sensitivity [1–3]. Furthermore, Kv1.3 inhibition has been proposed as a treatment for auto-immune diseases, obesity, neuroinflammation and other conditions [4–6]. In addition to its cell surface expression, this transmembrane protein is also located in the outer mitochondrial membrane where it has been linked to regulation of apoptosis and may therefore be a target for the treatment of cancer [7,8].

Voltage-gated potassium-selective channels displaying rapid activation and slow inactivation typical of Kv1.3 were first observed via patch clamp recordings in mammalian platelets by Maruyama[9]. Experiments in human platelets and murine megakaryocytes later demonstrated that these channels, encoded by *KCNA3*, are responsible for setting the resting membrane potential of approximately −50 to −60 mV [10–12]. Subsequent reports using megakaryocytes from other mammalian species further support these conclusions [13–15]. Kv1.3^−/-^ mice demonstrate that loss of the channel reduces platelet agonist-evoked Ca^2+^ responses and increases the circulating platelet count [11,16]. However, major questions remain regarding the overall impact of Kv1.3 on platelet responses and the underlying mechanisms. Using Kv1.3-deficient mice and a range of *in vitro* and *in vivo* assays, we have explored the contribution of this voltage-gated channel to platelet function and lifespan. A key finding is that Kv1.3 contributes to collagen-dependent adhesion and motility through interaction with the integrin α_2_β_1_. This advances our understanding of how Kv1.3 can contribute to function in platelets and other cell types, particularly within the immune system.

## Methods

### Reagents and antibodies

Antibodies for analysis of platelet surface antigens included FITC-conjugated rat anti-mouse GPIbα (CD42b, Xia.G5), GPIbβ (CD42c, Xia.C3), GPV (CD42d, Gon.C2) and rat anti-mouse isotype control (P190-1) from Emfret Analytics (Eibelstadt, Germany). Antibodies against integrin chains were FITC-conjugated α2 (CD49b, Ha 1/29), αIIb (CD41, MWReg30), β1 (CD29, Ha2/5), β3 (CD61, 2 C9.G2) and isotype controls from BD Biosciences (Wokingham, UK). Platelet α-granule secretion was measured using anti-*P*-selectin-FITC (CD62P, Wug.E9) and IgG isotype control, (Emfret Analytics). Horm collagen (type I fibrils from equine tendon) was obtained from Alere (Stockport, Cheshire, UK) and the collagen peptides CRP-XL: crosslinked GCO(GPO)_10_GCOG-amide, VWF-III: GPC(GPP_5_)GPRGQOGVMGFO(GPP)_5_GPC-amide, and GFOGER: GPC(GPP_5_)GFOGER(GPP_5_)GPC-amide, were from CambCol Laboratories (Ely, Cambs, UK). Fibrinogen, 3,3ʹ dihexyloxacarbocyanine iodide (DiOC_6_), prostaglandin E (PGE_1_), apyrase (type VII), ADP, and hirudin were all purchased from Sigma-Aldrich (Dorset, UK). FM®1-43 lipophilic styryl dye was from Molecular Probes (Life Technologies, Paisley, UK) and Phe-Pro-Arg-chloromethylketone (PPACK) from Hematologic Technologies Incorporated (Vermont, USA). DyLight® 649-conjugated anti-GPIbβ antibody (Emfret Analytics) was used for *in vivo* thrombus formation experiments.

### Animals and murine blood sampling

**Figure 1.**
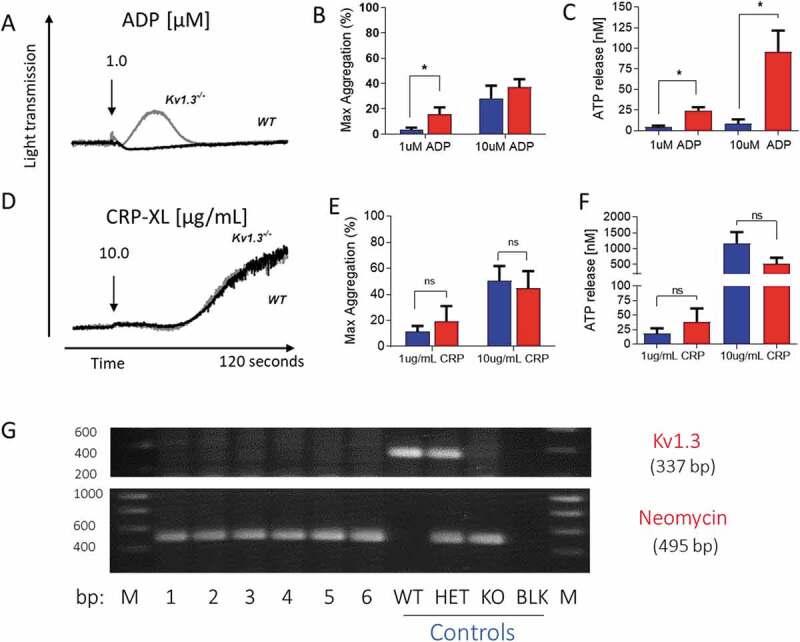
**Platelet aggregation and secretion in wild type and Kv1.3^−/-^ mice**. (A,D) Representative traces of platelet aggregation in response to (A) 1 µM ADP, and (D) 10 µg/mL CRP-XL (black line, C57BL/6 WT and gray line, Kv1.3^−/-^ (note that the WT and Kv1.3^−/-^ aggregation traces completely overlap in D). (B, E) Mean percent peak aggregation of washed murine platelets in response to ADP (1 and 10 μM, B) and CRP-XL (1 and 10 μg/mL, E) is shown for WT (blue) and Kv1.3^−/-^ (red) mice (mean ± SEM, n = 5). (C, F) Platelet dense granule secretion measured by analysis of ATP release in response to ADP (1 and 10 μM, C) and CRP-XL (1 and 10 μg/mL, F). Values are the mean ± SEM, n = 5; *P < .05, **P < .01, ns = not significant. (G) Representative gel showing the genotyping of C57BL/6 WT, heterozygous, and Kv1.3^−/-^ mice. WT display 337-bp WT band only, Kv1.3^−/-^ display the 495-bp neomycin band only, and heterozygous mice display both bands. The numbers across the bottom of the lanes denote individual samples and controls: bp = base pairs, M = molecular marker, lanes numbered 1–6 contain samples from Kv1.3^−/-^ mice, followed by control samples from wild type (WT), heterozygous (HET) and Kv1.3^−/-^ (KO) mice; BLK = PCR negative control, and M = molecular marker.

The generation of Kv1.3-deficient mice has been described previously[17]. These were backcrossed against C57BL/6 (Charles River, UK) and Kv1.3^−/-^ mice confirmed by genotyping (Figure 1). C57BL/6 mice matched for age and sex were purchased from Charles River, UK to represent wild-type (WT) controls. Experiments were carried out using mice of mixed gender. Blood was collected from the inferior vena cava of terminally isoflurane-anesthetised mice into 40 µM PPACK for whole blood *in vitro* studies of platelet adhesion under conditions of arterial flow, or acid citrate dextrose (ACD; 85 mM trisodium citrate, 78 mM citric acid and 111 mM glucose) for all assays using washed platelets (described below). All procedures were carried out in accordance with local and Home Office guidelines and approved Institutonal Animal Welfare Ethics Review Boards.

### Preparation of washed platelets

Whole blood drawn into ACD was centrifuged at 300 *g*, 3 minutes, the platelet-rich plasma (PRP) removed and re-centrifuged at 200 *g*, 2 minutes to pellet remaining red blood cells. The platelet suspension was supplemented with PGE_1_ (100 ng/mL) and apyrase (0.32 U/mL), and centrifuged at 1000 *g*, 10 minutes. The platelet pellet was washed in normal platelet saline (NPS: 145 mM NaCl, 5 mM KCl, 1 mM MgCl_2_, 10 mM *D*-glucose, 10 mM HEPES ~3.5 mM NaOH, pH 7.35) supplemented with 100 ng/mL PGE_1_ and apyrase (0.32 U/mL), and following centrifugation at 1000 *g*, was resuspended to original volume in NPS, and platelets adjusted to a concentration of 4 x 10^8^/mL.

### Flow cytometric analysis of platelet surface glycoproteins

Flow cytometry was used to measure the density of platelet surface glycoproteins. Washed platelets were diluted in NPS (1:10) and incubated for 15 minutes at room temperature with antibody or isotype control as per the manufacturer’s protocol. Platelet suspensions were diluted a further 1:10 in 0.2% formylsaline and analyzed by flow cytometer (BD Facscanto II; BD Biosciences, Wokingham, UK), gating the platelet population initially by size (Forward Scatter, FSC) and granularity (Side Scatter, SSC), followed by detection of mean fluorescent intensity of each surface antigen. All flow cytometry data was analyzed using Kaluza software (Beckman Coulter). Note that the values are reported as arbitrary fluorescence units since the signal depends upon the instrument gain and sensitivity.

### Aggregation and secretion studies

Turbidimetric measurement of platelet aggregation was performed using a model 400 lumi-aggregometer (Chronolog, Manchester, UK). Washed platelet suspensions were stirred for 3 minutes at 37°C, then fibrinogen (100 µg/mL), calcium chloride (2 mM) and agonist added (ADP or CRP-XL), and platelet aggregation recorded for 2 minutes. In parallel, ATP secretion from dense granules was measured using the CHRONO-LUME® luciferin;luciferase assay according to the manufacturer’s guidelines.

### Whole blood perfusion experiments

Whole blood was collected into 40 µM PPACK, and platelets loaded with 1 μM DiOC_6_for 20 minutes. Blood was perfused over coverslips coated with collagen (100 µg/mL), collagen peptide (100 µg/mL) or fibrinogen (200 µg/mL) at a shear rate of 1800 s^−1^ for collagen and collagen peptides, and 800 s^−1^ for fibrinogen. Thrombi were imaged by collection of a z-series of images acquired with an Olympus FV1000 confocal microscope at 3 separate fields per coverslip and analyzed in Image-J v1.49 (National Institutes of Health). Percent surface coverage, mean thrombus height and mean thrombus volume was calculated as described previously[18]. For study of platelet morphology, images of immobilized platelets were recorded at 30 minutes, and analyzed using Image-J.

### Platelet motility studies

Washed platelets were incubated with FM®1-43 (5 µM) and exposed to collagen-coated coverslips for 30 minutes in the absence of flow, and platelet movement and adhesion recorded in real-time on an Olympus FV1000 confocal microscope (excitation of FM®1-43 at 488 nm and emission at 550–650 nm). All experiments used a 60x oil immersion lens (UPLSAPO 60x, NA 1.35). The Image-J Manual Tracking plug-in was used to track the movement of platelets from each genotype as they attached and responded to the collagen fibers.

### In vivo thrombus formation

Thrombosis was measured in mouse cremaster arterioles as described previously[19]. Briefly, under general anesthesia the cremaster muscle was exteriorized and connective tissue removed. DyLight® 649-conjugated anti-GPIbβ antibody (0.2 µg/g mouse weight) was introduced into the carotid artery via a cannula. Injury to the vessel wall was made with a MicroPoint ablation laser (Andor Technology, Belfast, UK) and thrombus formation recorded using a digital camera with a charge-coupled device (C9300, Hamamatsu Photonics, Welwyn Garden City, UK). Data were analyzed using SlideBook 6 software (Intelligent Imaging Innovations, Denver, USA).

### Platelet survival

To determine platelet lifespan, 500 µg of biotin (EZ-link Sulfo NHS-SS-biotin, Thermo Scientific, Paisley, UK) was injected into the tail vein on Day 1. On each subsequent day, 50 µl of blood was collected by tail bleed into ACD. Following centrifugation at 125 *g* for 10 minutes, PRP was incubated with 20 µl streptavidin-APC (BD Biosciences) for 40 minutes at room temperature in the dark. The sample was washed with NPS and centrifuged for 6 minutes at 860 *g*, and the platelet pellet resuspended in 0.2% formylsaline for analysis by flow cytometry.

### Statistical analysis

All data and statistical analysis were performed using GraphPad Prism 6 (GraphPad Software, Inc, California, USA). Data are presented as mean ± SEM. For parametric data, comparison between 2 groups was performed using the Student *t* test and significance indicated as not significant (ns), *P ≤ .05, **P ≤ .01 and ***P ≤ .001.

## Results

### Expression of platelet surface glycoproteins

Deletion of Kv1.3 had no effect on expression of platelet surface glycoproteins GPIbα, GPIbβ, GPV, and integrin subunits α_2_, β_1_ and β_3_ (Table I). In contrast, integrin α_IIb_ was expressed at higher levels on platelets from Kv1.3^−/-^ compared to WT mice (*P* < .001). Since the α_IIb_ chain forms part of the α_IIb_β_3_ integrin complex, the main receptor for fibrinogen, we tested for possible differences in functional responses in which this major platelet adhesion ligand plays a key role, including aggregation, secretion and thrombus formation.

**Table I.** Platelet surface glycoprotein expression in WT and Kv1.3^−/-^ mice

	**WT mice** **(MFI)**	**Kv1.3^−/-^ mice** **(MFI)**	***P***
**GPIbβ**	14.48 ± 0.78	13.23 ± 0.95	.324
**GPIbα**	5.26 ± 0.51	4.39 ± 0.41	.202
**GPV**	2.16 ± 0.18	1.96 ± 0.24	.504
**α_2_**	0.95 ± 0.03	0.95 ± 0.01	.894
**β_1_**	6.36 ± 0.19	6.45 ± 0.38	.844
**α_IIb_**	4.82 ± 0.25	6.98 ± 0.44	**<.001**
**β_3_**	4.48 ± 0.12	4.63 ± 0.13	.438

Values minus the isotype control are given as the mean ± SEM. (n = 6–10).

MFI, mean fluorescent intensity (arbitrary units).

### Kv1.3^−/-^ platelets exhibit enhanced ADP-evoked aggregation and secretion

Absence of Kv1.3 was shown to promote platelet aggregation and secretion following P2Y receptor activation with ADP, but not stimulation of GPVI with the collagen peptide, CRP-XL. Platelets from Kv1.3^−/-^ mice displayed an elevated peak aggregation level in response to 1 µM ADP (*P* = .0395; Figure 1A, B), and significantly increased secretion of ATP from dense granules at 1 and 10 μM ADP (*P* = .0193; and *P* = .0204, respectively; Figure 1C). In contrast, aggregation and dense granule secretion were not significantly affected by loss of Kv1.3 in response to CRP-XL at either 1 μg/mL or 10 μg/mL (Figure 1D–F).

### Adhesion to fibrinogen and collagen under flow conditions

**Figure 2.**
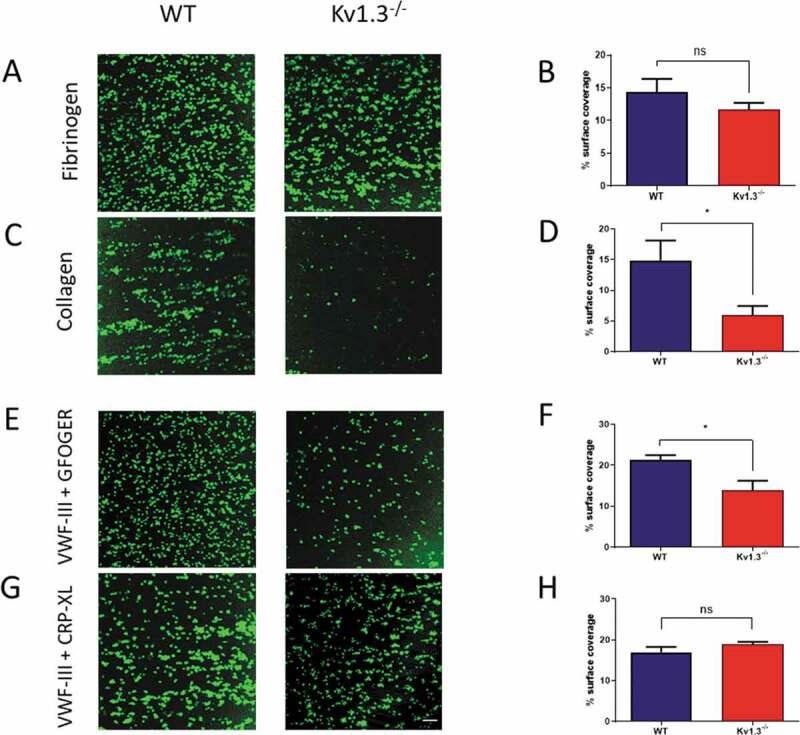
**Absence of Kv1.3 reduces integrin α_2_β_1_-dependent platelet adhesion to collagen**. DiOC_6_-labeled platelets in whole blood from WT or Kv1.3^−/-^ mice were perfused over fibrinogen (200 µg/mL) at a shear rate of 800 s^−1^ and collagen (100 µg/mL) at 1800 s^−1^. After 3 minutes of perfusion the coverslips were washed with normal platelet saline, and the images recorded and quantified as described in ‘Methods.’ Representative images (top panel) show platelet adhesion to fibrinogen (Figure 2A) and collagen (Figure 2C). Statistical analysis shows the percent of platelet surface adhesion (mean ± SEM) on fibrinogen (Figure 2B) and collagen (Figure 2D); (n = 5 for fibrinogen and 4 for collagen). DiOC_6_-labeled platelets in whole blood from WT or Kv1.3^−/-^ mice were also perfused at a shear rate of 1800 s^−1^ over coverslips coated with synthetic triple-helical peptides specific for the platelet collagen receptors integrin α_2_β_1_ (GFOGER, 100 µg/mL) and GPVI (CRP-XL, 100 µg/mL). Representative images (lower panel) show platelet adhesion to (E) peptides VWF-III and GFOGER, and (G) peptides VWF-III and CRP-XL. Scale bar = 20 µm. Statistical analysis shows the mean percent of platelet surface adhesion to (F) VWF-III and GFOGER (n = 5), and (H) VWF-III and CRP-XL (n = 5). (WT, blue; Kv1.3^−/-^, red). *P < .05, ns = not significant.

Platelet adhesion to immobilized fibrinogen was not significantly different following perfusion of blood extracted from Kv1.3^−/-^ compared to WT mice (*P* = .1209; Figure 2A, B). In contrast, flow-dependent platelet adhesion to fibrillar collagen was reduced in Kv1.3-deficient platelets (*P* = .0154; Figure 2C, D). Platelet adhesion to collagen under conditions of high/elevated shear is dependent initially on the transient engagement of the platelet GPIb complex with immobilized VWF, which facilitates subsequent direct interaction of platelet collagen receptors α_2_β_1_ and GPVI with the collagen fibrils, enabling firm adhesion to take place [20,21]. Under conditions of shear *in vitro*, platelets perfused over surfaces coated with VWF alone exhibit a rolling across the surface with only brief transient attachment[22]. Therefore we used the triple-helical collagen mimetic peptide, VWF-III, which contains the VWF-A3 binding motif that binds to collagen III, in combination with either the α_2_β_1_–specific peptide, GFOGER, or the GPVI-specific peptide, CRP-XL to investigate the contribution of Kv1.3 to platelet adhesion via the two collagen receptors under flow conditions. Platelets from Kv1.3^−/-^ mice had lower surface coverage on coverslips coated with VWF-III + GFOGER compared to platelets from WT mice (*P* = .0239; Figure 2E, F). In contrast, platelet surface coverage on coverslips coated with VWF-III + CRP-XL was not significantly different between the two genotypes (*P = *.2235; Figure 2G, H).

### Morphology of platelets adhering to collagen peptides

Adherent platelets were classified into four morphological categories representing different stages of platelet adhesion and activation[23]. Platelets that were rounded and without any protrusions, were classified as ‘round.’ Following initial adhesion, platelets begin to form protrusions to secure adhesion to the collagen fibrils, and these platelets were classified as ‘filopodia.’ Eventually, cytoskeletal remodeling allows actin fibers to fill between the filopodia, and platelets in this category were labeled as ‘ruffled’; and finally, depending on the matrix surface, platelets spread forming the typical ‘fried egg’ appearance.

**Figure 3.**
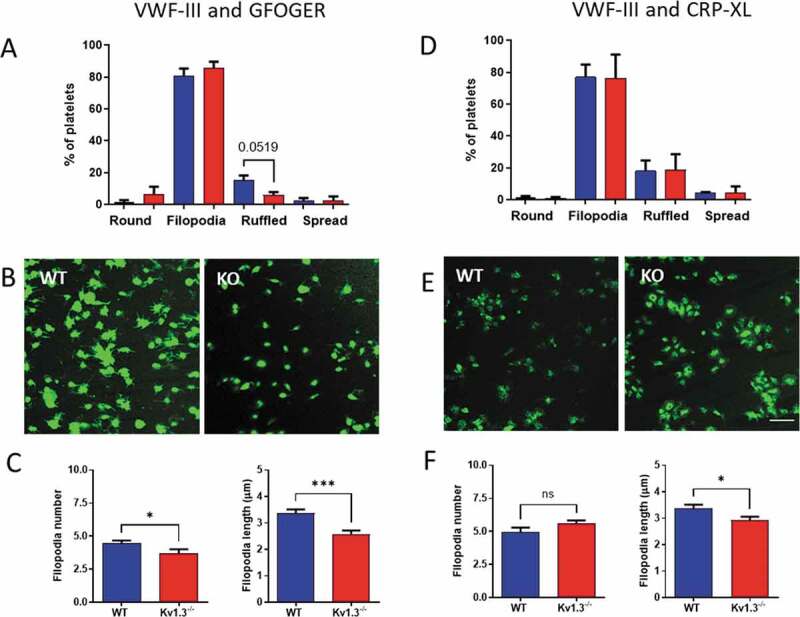
**Kv1.3-deficient platelets form fewer and shorter filopodia during integrin α_2_β_1_-dependent adhesion**. Platelet morphology following perfusion over collagen peptides at a shear rate of 1800 s^−1^, was classified as ‘round’ (round platelet with no protrusions), ‘filopodia’ (protrusions securing collagen fibrils), ‘ruffled’ (lamellipodia formation), and ‘spread’ (flattened or fried egg appearance). Distribution of platelet morphology following perfusion of WT and Kv1.3^−/-^ platelets over (A) VWF-III and GFOGER (100 µg/mL each peptide), and (D) VWF-III and CRP-XL (100 µg/mL each peptide). Data is expressed as the mean percent of platelets in each category (n = 5). Representative images of WT and Kv1.3^−/-^ platelet morphology on each peptide surface (B) VWF-III and GFOGER, and (E) VWF-III and CRP-XL. Scale bar = 10 µm. (C, F) Data shows the filopodia number per platelet (based on 50 platelets per treatment group), and filopodia length (µm)(based on length of 100 filopodia per treatment group) of WT and Kv1.3^−/-^platelets adhered to (C) VWF-III and GFOGER, and (F) VWF-III and CRP-XL. *P < .05, ***P < .005.

Following perfusion over the collagen peptides VWF-III and GFOGER a trend toward fewer Kv1.3^−/-^ platelets reaching the ruffled stage was observed, although this failed to reach significance (*P* = .0519; Figure 3A), and there was no difference in the percentage of platelets at each stage of platelet adhesion following perfusion over VWF-III and CRP-XL (Figure 3D). Interestingly, despite a similar number of platelets extending filopodia for the two genotypes, deletion of Kv1.3 resulted in a significant reduction in the number of filopodia (*P* = .0377) and also filopodia length (*P* = .003) for platelets adhered to VWF-III and GFOGER (Figure 3C). Representative images for platelets attached to VWF-III and GFOGER are shown in Figure 3B and for VWF-III and CRP-XL in Figure 3E.

### Motile responses of platelets adhering to collagen

**Figure 4.**
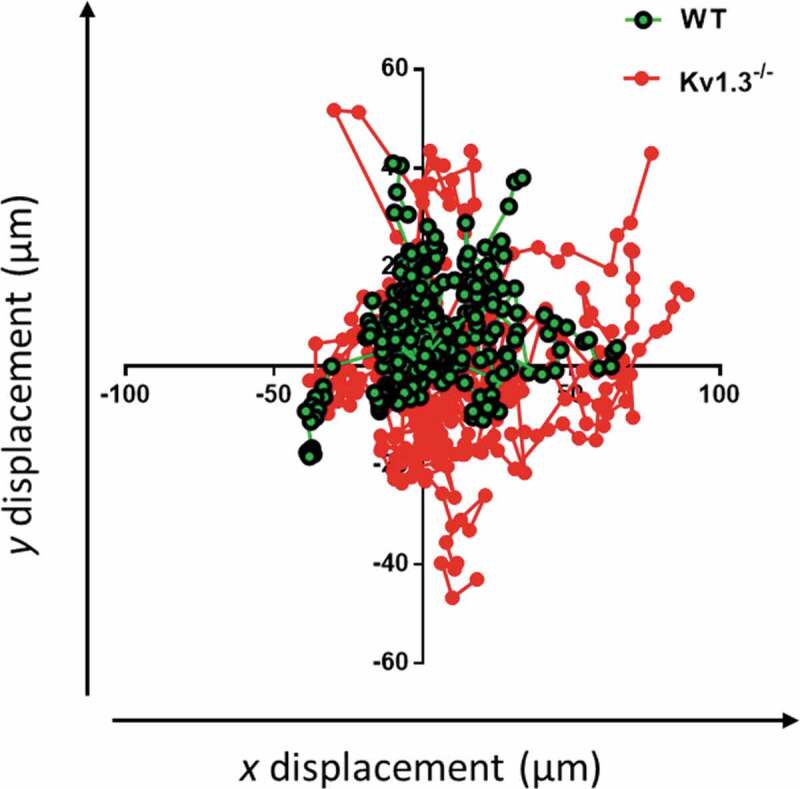
**Kv1.3-deficient platelets lack directional persistence during adhesion to collagen via α_2_β_1_**. The morphology and motile responses of murine platelets labelled with FM®1-43 lipophilic styryl dye (5 µM) were tracked during adhesion to collagen (100 µg/mL) under static conditions. The plotting of co-ordinates tracked the trajectories of WT and Kv1.3-deficient platelets during platelet attachment to the collagen fibers. Platelet movement and direction is measured by displacement (µm) along the x and y axis. Data from 3 independent experiments.

To further assess the effect of the altered morphology of Kv1.3^−/-^ platelets during integrin α_2_β_1_-dependent platelet adhesion, the motile responses of WT and Kv1.3^−/-^ platelets were tracked during adhesion to fibrillar collagen under static conditions. The tracked trajectories of individual Kv1.3^−/-^ platelets traveled over a wider area than WT platelets (Figure 4A). Observation of video recordings of WT and Kv1.3^−/-^ platelet movement supported these findings of an altered Kv1.3^−/-^ platelet motile response to collagen. During initial attachment to collagen, platelets are able to extend long filopodia toward the collagen fibrils (Supplemental video 1). We observed that WT platelets (Supplemental video 2), rapidly extrude filopodia and pull on the collagen fibers to firmly adhere. In contrast, Kv1.3-deficient platelets displayed a loss of directional persistence, with fewer long filopodia and reduced pulling on collagen fibers (Supplemental video 3).

### In vitro thrombus formation on collagen

**Figure 5.**
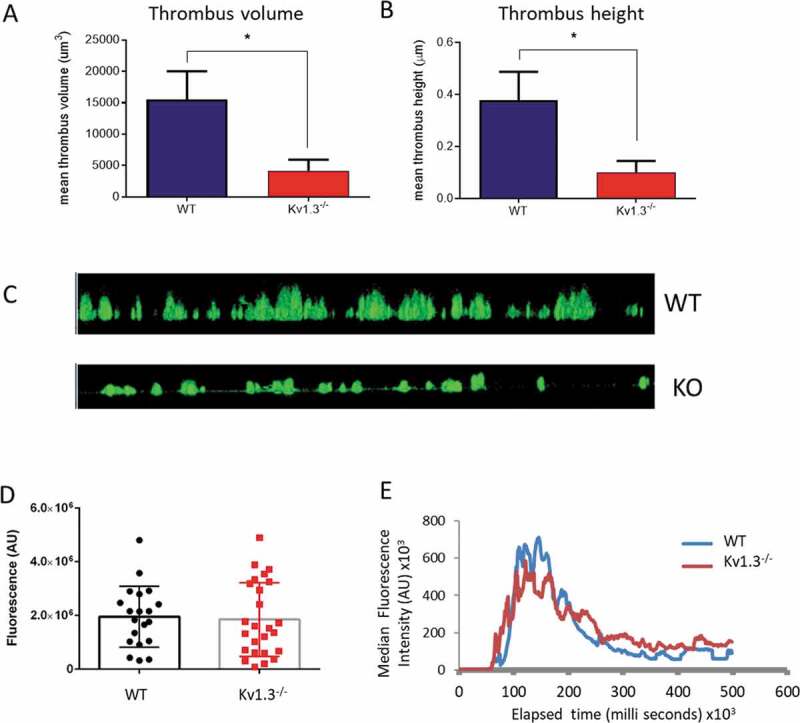
**Absence of Kv1.3 reduces *in vitro*, but not *in vivo*, thrombus formation**. DiOC_6_-loaded platelets from Kv1.3^−/-^ mice were perfused over collagen-coated coverslips (100 µg/mL), and analyzed for (A) total thrombus volume (µm^3^) and (B) thrombus height (µm). Data shown is the mean ± SEM for thrombi formed by platelets from WT (blue) and Kv1.3^−/-^ mice (red) (n = 4; *P < .05. (C) Representative side elevation of z-stack fluorescent images of thrombi formed on fibrillar collagen by platelets from WT (upper image) and Kv1.3^−/-^ (lower image) mice. (D) Mean integrated fluorescence (arbitrary units), and (E) Plot of median fluorescence intensity (arbitrary units) over time (seconds), during *in vivo* thrombus formation following laser-induced injury in cremaster muscle arterioles of WT and Kv1.3^−/-^ mice; (*n* = 20 thrombi in 5 WT mice and 25 thrombi in 5 Kv1.3^−/-^ mice).

The above experiments demonstrate that deletion of platelet Kv1.3 results in diminished responses involving integrin α_2_β_1_, such as adhesion to collagen, but that α_IIb_β_3_-dependent functions, such as aggregation, are not reduced. This suggests that Kv1.3^−/-^ platelets should be able to aggregate and form thrombi where platelet attachment to collagen has been successful. Following platelet perfusion over collagen at a shear rate of 1800 s^−1^, Kv1.3-deficient platelets that did successfully adhere to collagen fibrils were able to form thrombi, but with significantly reduced height (*P* = .0254; Figure 5A), and volume (*P = *.0254; Figure 5B). The difference between thrombi formed by WT and Kv1.3^−/-^ platelets on fibrillar collagen can also be seen in the side elevation of z-stack fluorescent images in Figure 5C.

### Characterization of platelet function in vivo: thrombus formation

To investigate whether the altered *in vitro* responses of Kv1.3^−/-^ platelets translate into a change in function within the circulation, we studied thombus formation in the cremaster muscle arterioles of anaesthetised male mice using the laser-induced injury model [24–26]. The profile of median thrombus fluorescence (*n* = 20 thrombi in five WT mice and 25 thrombi in five Kv1.3^−/-^ mice) through the 500 second duration of thrombus growth and regression in Kv1.3-deficient mice was not significantly different from thrombi formed in WT mice (Figure 5E). Consistent with this, maximum thrombus fluorescence was not altered (Figure 5D).

### Platelet size and platelet lifespan

**Figure 6.**
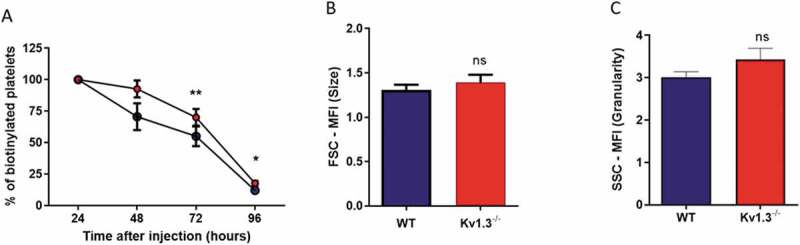
**Platelets from Kv1.3^−/-^ display a longer lifespan**. (A) Assessment of platelet lifespan was carried out using *in vivo* biotinylation of murine platelets, recording the percent of biotinylated platelets isolated from WT and Kv1.3^−/-^ mice over five days (n = 4 for each genotype). Flow cytometric analysis of (B) platelet size and (C) platelet granularity gating on forward scatter and side scatter of platelet populations isolated from WT and Kv1.3^−/-^ mice (n = 8). Data shown is mean ±SEM. **P < .01, *P < .05, ns not significant.

Kv1.3-deficient mice display elevated platelet counts, but no change in megakaryocyte development within the marrow[11]. To explore whether an altered lifespan could account for this phenotype, we measured platelet clearance in Kv1.3^−/-^ and WT mice using an *in vivo* biotinylation approach[27]. The percent biotinylated platelets was significantly elevated above that measured in the platelet population from WT mice at 72 hours (*P* = .006) and at 96 hours (*P* = .0452) post biotin injection (Figure 6A), suggesting that an enhanced lifespan may contribute to the greater platelet count observed in Kv1.3^−/-^ mice. Kv1.3^−/-^ platelets were no different in size (*P* = .1979; Figure 6B), or granularity (*P* = .198; Figure 6C), as determined by Forward and Side Scatter using flow cytometry.

## Discussion

Kv1.3 plays a crucial role in maintaining the resting membrane potential in platelets [10,11], regulating entry of the key second messenger Ca^2+^; however, its contribution to hemostasis and thrombosis is less clear. The present study provides new evidence that loss of Kv1.3 in murine platelets modulates a number of platelet responses, particularly collagen-evoked adhesion and motile responses through a mechanism dependent on integrin α_2_β_1_.

Platelet adhesion and thrombus formation relies on two key integrins, namely α_2_β_1_ which binds to collagen, and α_IIb_β_3_ which binds several ligands including fibrinogen, fibronectin, Von Willebrand factor (VWF), and fibrin[28]. Our studies of murine platelet adhesion under conditions of arterial shear demonstrate that adhesion to fibrillar collagen, where α_2_β_1_ is an important adhesive receptor, is significantly impaired following deletion of Kv1.3. In contrast, loss of Kv1.3 had no effect on α_IIb_β_3_-dependent platelet binding to immobilized fibrinogen. This specific role for Kv1.3 in α_2_β_1_-mediated adhesion was further demonstrated in experiments using combinations of triple-helical collagen-specific peptides. Kv1.3^−/-^ platelet adherence was significantly reduced compared to those from WT mice when perfused over surfaces coated with a combination of VWF-III, a peptide which contains the VWF-A3 collagen binding motif, and the integrin α_2_β_1_-specific peptide, GFOGER. In contrast, no phenotypic difference was observed between adhesion of WT and Kv1.3^−/-^ platelets perfused over surfaces coated with VWF-III combined with the GPVI-specific collagen peptide, CRP-XL. This is consistent with experiments using human platelets, where Pugh and colleagues used integrin-specific peptides of differing affinity to show that α_2_β_1_ enhances the rate of recruitment of platelets to a collagenous surface[29].

Exposure to collagen induces marked changes in platelet morphology. Initial events involve the extension of filopodial protrusions which allow attachment to collagen fibers, followed by formation of actin-rich lamellipodia and eventual transformation to the typical ‘fried egg’ appearance during platelet spreading [23,30]. Mutations that affect the molecular mechanisms and protein interactions of cytosketeletal reorganization during platelet shape change impair the ability of platelets to adhere and form a thrombus [31–34]. Our studies of DiOC_6_-loaded platelets demonstrate that in the absence of Kv1.3^−/-^, fewer platelets progress to form lamellipodia (ruffled appearance) on α_2_β_1_-dependent surfaces coated with VWF-III/GFOGER; furthermore, Kv1.3^−/-^ platelets exhibited shorter and fewer filopodia compared to WT controls. Video recordings under static conditions show that WT platelets extend long filopodia toward collagen fibers, pulling the fibers upon initial attachment, before spreading (Supplementary video 1 and 2), whereas platelets lacking Kv1.3 are less able to facilitate attachment (Supplementary video 3). Subsequent tracking of the trajectories of individual platelet movement under the same conditions further demonstrated that Kv1.3^−/-^ platelet haptotaxis toward collagen is less efficient, with platelets traveling over a wider area, appearing to have less ability to ‘sense’ the collagen or maintain directional persistence toward it. This is consistent with weaker integrin-dependent adhesion permitting greater motility, as shown in HT1080 fibrosarcoma haptotaxis experiments[35]. Interestingly, blockade or reduced expression of Kv1.3 also impairs migration of T-lymphocytes [36,37], and alters detection of electrical fields in neutrophils[38]. Voltage-gated sodium channels (Na_v_) have also been proposed to contribute to cellular motility and migration in several types of immune cells including lymphocytes [39] and macrophages [40] and intracellularly localized Na_v_1.6 supports invasiveness of human breast cancer cells[41]. Sodium channel β subunits are crucial components of the mechanism whereby pore-forming α subunits Na_v_1.5 or 1.6 regulate adhesion and migration and there is evidence that Na_v_β_1_ can act independently as a cell adhesion molecule [42–44].

Thrombus formation by DiOC_6_-labeled Kv1.3^−/-^ platelets is inhibited during perfusion over collagen-coated surfaces. Our results suggest that this may be due to a Kv1.3-associated contribution to the formation of platelet filopodia and their mechanosensing ability to detect collagen fibrils in the microenvironment, rather than a defect in platelet aggregation. Surprisingly, however, we saw no difference in thrombus formation or thrombus size in cremaster muscle arterioles of WT or Kv1.3^−/-^ mice following laser injury using an *in vivo* model that causes endothelial damage and collagen exposure[45]. A similar lack of arterial thrombosis phenotype in Kv1.3^−/-^ mice has also recently been reported using alternative models of thrombosis which depend more upon activation by collagen than the laser injury model used in the present study [16]. Therefore, the lack of Kv1.3 iscompensated for *in vivo* by other pathways. The enhanced aggregation and secretion that we observed *in vitro* with ADP in Kv1.3^−/-^ platelets may explain this compensation. We observed increased α_IIb_ integrin expression on the surface, however β_3_ subunit expression was not altered. It is possible that the α_IIb_ subunit is expressed on the surface independently of associated beta subunits; this is known to happen for other integrin subunits [46]. In its monomeric form it would not contribute to enhanced aggregation unless it can combine with other beta subunits, which requires further study. Although Fan and collagues [16] have observed an upregulation in expression of KCNQ4 and other K^+^ channels in Kv1.3^−/--^ platelets, we observed no direct evidence for such a change in our previous electrophysiological recordngs [11]. It is known that that secondary activation of P2Y12 receptors by released ADP amplifies collagen-evoked platelet aggregation [47,48]. Thus, the enhanced ADP responses in Kv1.3-deficient platelets may contribute to the lack of significant difference in collagen-evoked aggregation in standard stirred suspension. Given this argument, the reduced thrombus formation under arterial shear is somewhat unexpected. However, the altered motile responses to collagen may be the overriding determinant of whether the platelets initially attach and therefore can generate a thrombus.

We previously reported increased platelet count in Kv1.3^−/-^ mice which was not due to an altered frequency or size of bone marrow MKs[11]. Here we show that significantly higher numbers of biotinylated platelets remained in the circulation of Kv1.3^−/-^ mice post-injection compared to WT. Platelet lifespan in the mouse is around 5 days, and is regulated by components of the intrinsic apoptotic pathway, whereby the pro-survival protein family member, Bcl-xL controls the activity of pro-apoptotic proteins Bak and Bax [49,50]. Studies in lymphocytes have identified Kv1.3 on the inner mitochondrial membrane[51], where it plays a role in the induction of apoptosis through its interaction with Bax [52,53]. Further study is needed to confirm the possible existence and potential contribution of mitoKv1.3 to platelet apoptosis, but it is possible that platelet apoptosis is impaired when Bax cannot interact with mitoKv1.3. This potential role for Kv1.3 in platelet production, and pro-survival phenotype in the absence of Kv1.3, may contribute to elevated levels of platelets in the circulation of the Kv1.3^−/-^ mice. The recent study by Fan and colleagues [16] confirmed the increased platelet count phenotype of Kv1.3^−/-^ mice. without a change in marrow megakaryopoiesis, and additionally demonstrated increased numbers of megakaryocytes in the spleen. This organ is an alternative site of platelet production in the mouse as well as a site of platelet clearance, and thus may also contribute to the increased platelet count following Kv1.3 deletion. The elevated megakaryopoiesis and reduced clearance is likely to increase the number of reticulated platelets in the circulation, which are known to be more reactive than mature platelets [54,55], and this may partially explain the enhanced aggregation and secretory response to low concentrations of ADP in Kv1.3^−/-^ platelets. Although Fan and colleagues [16] concur with a lack of *in vivo* thrombus formation phenotype of Kv1.3^−/-^ mice, they report that aggregation of platelets to several agonists *in vitro*, including thrombin and collagen, and high dose ADP (20 µM), are reduced by loss of channel function following either genetic deletion or application of a pore-blocking antibody (6E12#15)[16]. The difference between the two studies requires further investigation but may result from variability in the method of preparing platelets for *in vitro* studies.

The experiments reported here raise a number of key questions that we have not been able to investigate due to the impact of the COVID-19 pandemic on our laboratories. This challenge has been recognized by Journal editorial policies [56]andwe report here the completed aspects to the work which highlight the need for additional studies. Future experiments should investigate the mechanism responsible for enhanced ADP-evoked aggregation and secretion in Kv1.3^−/-^ platelets and whether responses to other G-protein-coupled receptor agonists are affected. We also propose a pharmacological approach using blockers such as margatoxin and Pap-1. A key area to investigate is the mechanism by which Kv1.3 modulates integrin function and motility; particularly important questions are whether channel opening is required and whether there is an involvement of the K^+^ channel regulatory proteins identified in our platelet ion channel transcriptome study[12]. The effect of Kv1.3 deletion on interactions with other adhesive substrates is worthwhile investigating, which would benefit from more advanced imaging approaches. Given the enhanced platelet lifespan and increased platelet number in Kv1.3-deficient mice, additional studies should also investigate the presence of mitoKv1.3 and its potential role in the platelet.

Although platelets are highly specialized for hemostasis, they also contribute to immune responses, often serving as a link between the hemostatic and inflammatory systems [57–60]. For example, they facilitate the phagocytic removal and sequestering of pathogens [61–63] and release antimicrobial agents and chemokines [64,65]. Kv1.3 has a well established role in immune function, particularly in T-lymphocytes [66–68] and its overexpression is a common feature of chronic inflammatory diseases, contributing to the over-reaction of cellular immunity and subsequent cytokine storm [69,70]. Interestingly, in a study using the middle cerebral artery occlusion model, a model of ischemic stroke that involves the formation of occlusive platelet thrombi in response to combined thrombotic and inflammatory stimuli[71], the selective Kv1.3 blocker Pap-1 dose-dependently reduced the infarct area in rodents, reducing microglial activation and improving neuronal survival[72]. In light of the present study and work by Fan and colleagues[16], it is worthwhile exploring the relative contribution of platelet Kv1.3 to the etiology of immune disorders.

## Supplementary Material

Supplemental Material

Supplemental Material

Supplemental Material

## Key points

- The voltage-gated K^+^ channel Kv1.3 enhances collagen-evoked platelet adhesion and thrombus formation through an α_2_β_1_ integrin-dependent mechanism
- Kv1.3-deficient platelets display reduced filopodia formation and greater motility during attachment to collagen fibres
